# An Electrocorticographic Brain Interface in an Individual with Tetraplegia

**DOI:** 10.1371/journal.pone.0055344

**Published:** 2013-02-06

**Authors:** Wei Wang, Jennifer L. Collinger, Alan D. Degenhart, Elizabeth C. Tyler-Kabara, Andrew B. Schwartz, Daniel W. Moran, Douglas J. Weber, Brian Wodlinger, Ramana K. Vinjamuri, Robin C. Ashmore, John W. Kelly, Michael L. Boninger

**Affiliations:** 1 Department of Physical Medicine and Rehabilitation, University of Pittsburgh, Pittsburgh, Pennsylvania, United States of America; 2 Department of Bioengineering, University of Pittsburgh, Pittsburgh, Pennsylvania, United States of America; 3 Clinical and Translational Science Institute, University of Pittsburgh, Pittsburgh, Pennsylvania, United States of America; 4 Center for the Neural Basis of Cognition, Carnegie Mellon University and the University of Pittsburgh, Pittsburgh, Pennsylvania, United States of America; 5 Human Engineering Research Laboratories, Department of Veterans Affairs, Pittsburgh, Pennsylvania, United States of America; 6 Department of Neurological Surgery, University of Pittsburgh, Pittsburgh, Pennsylvania, United States of America; 7 Department of Neurobiology, University of Pittsburgh, Pittsburgh, Pennsylvania, United States of America; 8 Departments of Biomedical Engineering and Neurobiology, Washington University in St. Louis, St. Louis, Missouri, United States of America; 9 Department of Electrical and Computer Engineering, Carnegie Mellon University, Pittsburgh, Pennsylvania, United States of America; Emory University, United States of America

## Abstract

Brain-computer interface (BCI) technology aims to help individuals with disability to control assistive devices and reanimate paralyzed limbs. Our study investigated the feasibility of an electrocorticography (ECoG)-based BCI system in an individual with tetraplegia caused by C4 level spinal cord injury. ECoG signals were recorded with a high-density 32-electrode grid over the hand and arm area of the left sensorimotor cortex. The participant was able to voluntarily activate his sensorimotor cortex using attempted movements, with distinct cortical activity patterns for different segments of the upper limb. Using only brain activity, the participant achieved robust control of 3D cursor movement. The ECoG grid was explanted 28 days post-implantation with no adverse effect. This study demonstrates that ECoG signals recorded from the sensorimotor cortex can be used for real-time device control in paralyzed individuals.

## Introduction

Brain-computer interface (BCI) technology aims to establish a direct link for transmitting information between the brain and external devices [1]–[4]. It has the potential to improve the quality of life for individuals with disability as it may offer a natural and rich control interface for assistive devices [5]–[11]. Key criteria to realize a clinically-viable BCI device include the ability to record neural activity with high spatial and temporal resolution, reliability for long-term use with substantial functional benefit, minimal invasiveness, and the potential to operate autonomously. Electrocorticography (ECoG) measures cortical field potentials using electrodes placed on the surface of the brain, and used carefully, can satisfy each of these criteria [12]–[14]. Work with patients undergoing clinical brain mapping, e.g. for seizure or pain treatment, has demonstrated that BCI control signals can be extracted from ECoG [7], [12], [15]–[20]. The current study investigated the feasibility of an ECoG-based BCI system in an individual with tetraplegia caused by spinal cord injury. A high-density ECoG grid was implanted subdurally over this individual's sensorimotor cortex for 28 days, during which the individual was trained to control 2D and 3D cursor movement using ECoG signals.

## Materials and Methods

### Ethics Statement

This study was approved by the Institutional Review Board at the University of Pittsburgh and followed all guidelines for human subject research. Written informed consent was obtained before initiating any research procedures (Text S1, Supplementary Note 1). The individuals in this manuscript have given written informed consent (as outlined in PLOS consent form) to publish these case details and videos.

### Study Participant

The participant was a 30-year-old right-handed male with tetraplegia caused by a complete C4 level spinal cord injury [21] seven years prior to the experiment. The participant had no volitional arm or hand movement.

### High-Density ECoG Grid

**Figure 1:**
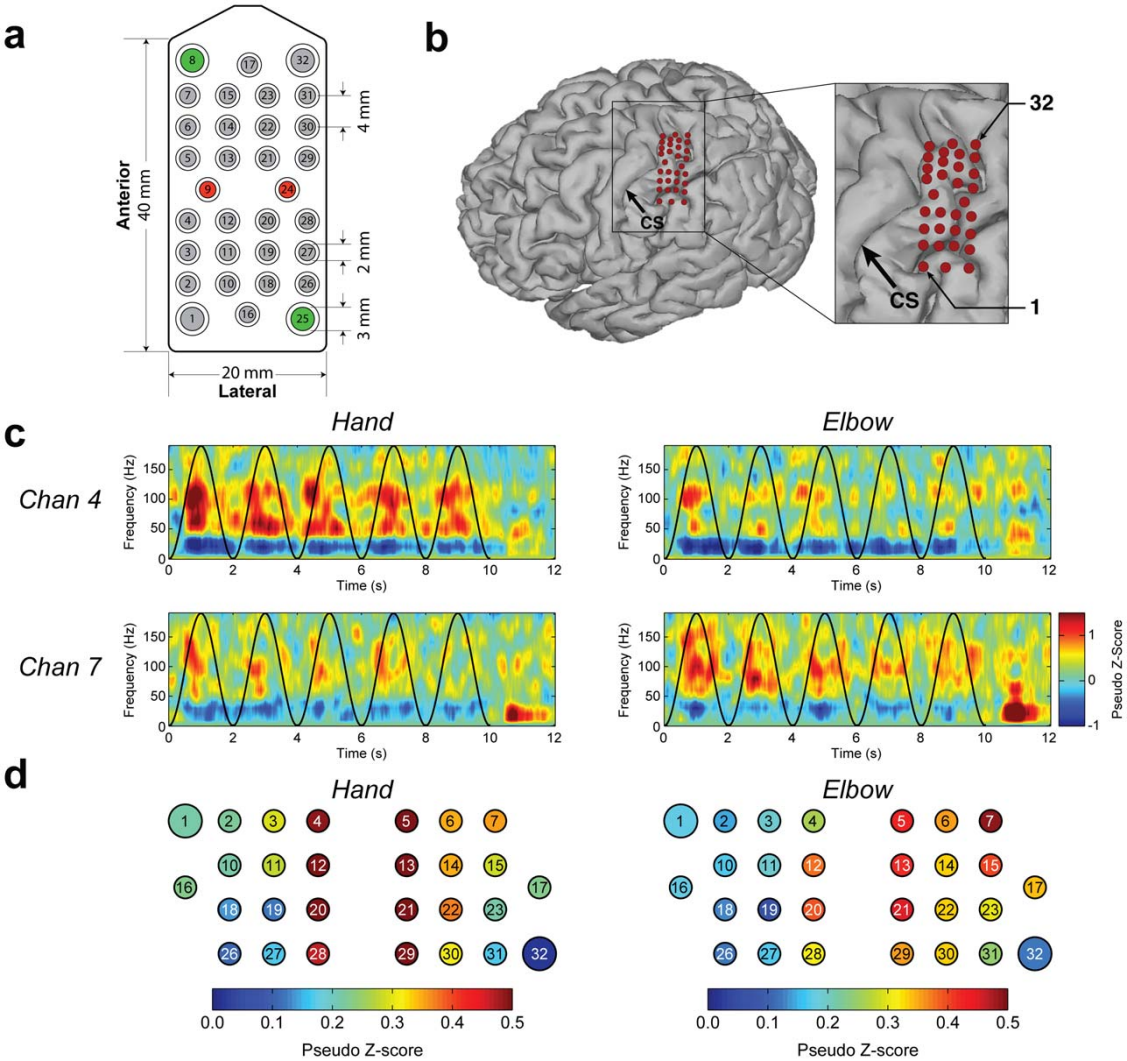
High-density ECoG grid location and ECoG signal modulation during motor screening tasks. (a) Layout of the recording (gray, brain-facing), reference (red, dura-facing), and ground (green, dura-facing) electrodes. (b) ECoG electrode location mapped to a 3D rendering of the participant's brain. Red dots represent ECoG electrodes, and Electrodes 1 and 32 are labeled to indicate grid orientation. The black arrow indicates the central sulcus (CS) of the left hemisphere. (c) Modulation of ECoG signals by attempted hand opening/closing (left column) and elbow flexion/extension (right column) for Channel 4 (top row) and Channel 7 (bottom row). These four time-frequency plots show data averaged over 24 trials. Black sinusoidal curves overlaid on all plots represent the normalized instructed joint angles. Time 0 is the onset of visual cues (hand fully-open, elbow fully-extended). Color represents pseudo z-scores, indicating changes from baseline condition, and color axes of all plots have the same range. Red and blue colors indicate increases and decreases in spectral power, respectively. High-gamma band (70–110 Hz) powers increased for Channels 4 and 7 during attempted hand and elbow movements, respectively. Also, for both channels, the high-gamma band power differed between attempted hand and elbow movements. (d) Cortical activity patterns across all 28 recording electrodes during attempted hand and elbow movements represented by 70–110 Hz band power over the 10-second movement time averaged across 24 trials. The color bars represent pseudo z-scores. Cortical activity patterns differed between hand and elbow movements.

The custom ECoG grid (PMT Corp, Chanhassen, MN USA) was composed of a silicone sheet (2 cm×4 cm in size, 1 mm thick) and 32 platinum disc electrodes with 28 recording electrodes facing the brain and 4 ground and reference electrodes facing the dura (**Fig. 1A**). Electrodes were either 2 or 3 mm in diameter and were spaced 4 mm apart. Platinum lead wires from all electrodes formed two 60 cm long leads with 32 standard ring connectors for ECoG recording.

### Presurgical and Surgical Procedures

Six weeks prior to the implantation surgery, functional magnetic resonance imaging (fMRI) was conducted while the participant watched videos of hand and arm movement and attempted the same movement in order to localize the left sensorimotor cortex and guide the grid placement. The participant also went through standard presurgical screening including physical examination, blood and urine analysis, and chest x-ray. On Day 0 (August 25, 2011), the ECoG grid was implanted subdurally over the hand and arm areas of left sensorimotor cortex (**Fig. 1B**) (Text S1, Supplementary Note 2). Two leads of the ECoG grid were tunneled subcutaneously to the chest to pass through the skin below the left clavicle. A sterile dressing covered the exit site, and the leads were physically connected to the neural recording system during experiment sessions. The participant returned home after an overnight hospital stay, with testing commencing on Day 2 post-op. On Day 28, the grid was explanted following the U.S. Food and Drug Administration regulation. Electrode locations on the subject's brain surface were determined using post-operative head x-ray and computed tomography (CT) images along with coordinates of exposed electrodes recorded by the surgical navigation system (Brainlab AG, Feldkirchen, Germany) during the grid implantation surgery (Text S1, Supplementary Note 3) [22], [23].

### Motor Screening and BCI Tasks

Over the 28-day period, the study testing occurred at the participant's home (12 days) and the research lab (9 days), and the participant had six days to rest and conduct personal activities. The participant performed multiple motor screening and BCI tasks (**Fig. S1**). First, during the motor screening task, the participant observed right hand and arm movements of a virtual character on an LCD screen and simultaneously attempted the same movement. Second, the spectral power in the 70–110 Hz frequency band of each ECoG electrode was shown in real-time to the participant, allowing the researchers and participant to find specific attempted movements that consistently elicited distinct patterns of cortical activity across the grid. Third, the participant controlled a cursor in a virtual environment in real-time using ECoG signals performing two- and three-dimensional (2D and 3D) center-out tasks [8], [24]. The timeout periods were 5 and 7 seconds for the 2D and 3D tasks, respectively, and a trial was considered successful once the cursor touched the target. The cursor center was constrained within the workspace boundary. The virtual environment used a Cartesian coordinate system where the x-axis pointed to the subject's right, the y-axis pointed upward, and the z-axis pointed toward the subject. On Day 27, the participant attempted to control 3D movement of a dexterous prosthetic arm (The Applied Physics Laboratory, Laurel, MD, USA) _ENREF_43 [25] to reach for objects and other individuals' hands. This was intended only as a brief demonstration since a more extensive study was precluded by the limited duration of the protocol.

### Neural Signal Decoding and BCI Control Schemes

Twenty-eight channels of ECoG signals were recorded with the g.USBamp biosignal amplifier (Guger Technologies, Austria). Craniux, LabVIEW-based open-source BCI software developed in our laboratory, was used for signal processing, neural decoding, and experiment control [26]. The g.USBamp sampled raw ECoG signals at 1200 Hz and sent a block of real-time ECoG data to the Craniux software every 33 ms, leading to a system update rate of 30 Hz. For convenience of discussion, we define an ECoG signal feature as the power of one 10 Hz wide frequency band from one channel. The Craniux software calculated the power in twenty 10 Hz wide frequency bands between 0 to 200 Hz for each of 28 channels in real-time using 25^th^ order auto-regressive (AR) estimation [27] over a 300 ms window every 33 ms. For each channel, the spectral power for each frequency band was then log-transformed and converted to a pseudo z-score (i.e. instantaneous feature activity) using the mean and standard deviation of the same band's log-transformed power during the baseline resting condition [28], [29]. Real-time BCI control used 448 ECoG signal features (sixteen 10 Hz wide bands between 40–200 Hz) encompassing the gamma and high-gamma bands across 28 channels.

The neural decoder of the BCI system transformed instantaneous feature activities (***f***) into 2D or 3D cursor velocity control signals (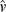) in real-time based on Equation 1. The decoding weights (*W*) were calculated using the optimal linear estimator (OLE) algorithm [30], [31] based on Equation 2 (Text S1, Supplementary Note 5): 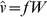
(1)

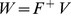
(2)where *V* and *F* are matrices representing the desired cursor movement direction and associated feature activities. The desired cursor movement direction is the unit vector pointing from the cursor to the target. The superscript “+” denotes the pseudo-inverse of a matrix.

In order for the participant to systematically modulate cortical activity for BCI control, the participant was instructed to associate attempted movement with desired cursor movement direction (**Fig. S2**). For controlling 2D cursor movement within the x-y plane, the participant associated four attempted flexion/extension movement patterns with four cursor movement directions: thumb (left), elbow (right), both thumb and elbow (up), and no thumb or elbow movement (down) (Text S1, Supplementary Note 6). Additionally, for the 3D task, attempted wrist flexion and extension were used to move the cursor in the positive z-direction, and the cursor moved in the negative z-direction when there was no attempted wrist movement. By instructing the participant to associate desired cursor movement direction with attempted thumb, elbow, and wrist movements, we aimed to link desired cursor movement direction to ECoG signal modulation, which would enable the OLE decoder to directly extract cursor velocity control signals from ECoG (**Fig. S2** and **Equations 1** **and** **2**). This control scheme enabled cursor movement in arbitrary directions, at variable velocity in all three dimensions simultaneously (Text S1, Supplementary Note 6).

This study used a “turn-taking adaptation” scheme (**Fig. S2**) where the adapting agent was alternated between the human subject and the neural decoder of the BCI system (Text S1, Supplementary Note 7). During the human adaptation period, the neural decoding weights were held constant while the participant adjusted his attempted movements and control strategy based on real-time feedback of brain-controlled cursor movement to improve control accuracy, i.e. “the subject learns the decoder”. During the computer adaptation period, the participant was instructed to perform the same attempted movements repetitively without correcting for errors in the brain-controlled cursor movement. Meanwhile, neural decoding weights were updated periodically, i.e. “the decoder learns the subject” [32]. Furthermore, the transition from 2D to 3D control was conducted by gradually blending decoding weights calculated for the 3D task into the existing 2D decoding weights using the turn-taking adaptation scheme. Finally, computer assist was used to facilitate brain control training. The assistance attenuated the component of the cursor control signal perpendicular to the vector from the cursor to the target by an experimenter-controlled percentage [9]. At 100% computer assist, the cursor will stay on a straight trajectory from the center of the screen toward the target. At 0% computer assist, there will be no constraint on cursor movement in any direction. Such computer assist was used to reduce task difficulty during initial BCI training.

### Characterization of Brain-Controlled Cursor Movement

The distance ratio was calculated as the actual trajectory length divided by the length of an ideal straight-line path [33]. Movement error was calculated as the average perpendicular distance between the cursor and the ideal straight-line path normalized by the distance between the center and the peripheral targets [33]. Additional metrics were time to target and percent time at the boundary, i.e. the number of time points when the cursor center was touching the workspace boundary divided by the total number of time points during a certain number of brain control trials. The chance success rate was determined by reconstructing cursor movement from recorded ECoG signals and randomly-shuffled decoding weights; this process was repeated 10,000 times. The 3D task was performed in 80-trial blocks with resting between blocks; data from the last block were used to characterize the final 3D cursor control performance.

## Results

### Cortical Activity during Motor Screening

ECoG signals recorded from the left sensorimotor cortex demonstrated modulation when the participant observed and simultaneously attempted right hand and arm movement even though the participant was unable to generate overt movements. The most prominent modulation patterns were an increase in power for the gamma and high-gamma bands and a decrease in power for the sensorimotor rhythm (10–30 Hz), both tightly coupled in time with the movement (**Fig. 1C**). Attempted movements of hand and elbow elicited distinct cortical activity patterns, with the centers of activation being lateral for attempted hand movement and medial for attempted elbow movement on the ECoG grid (**Fig. 1D**).

### Cortical Control of 2D Cursor Movement

**Figure 2:**
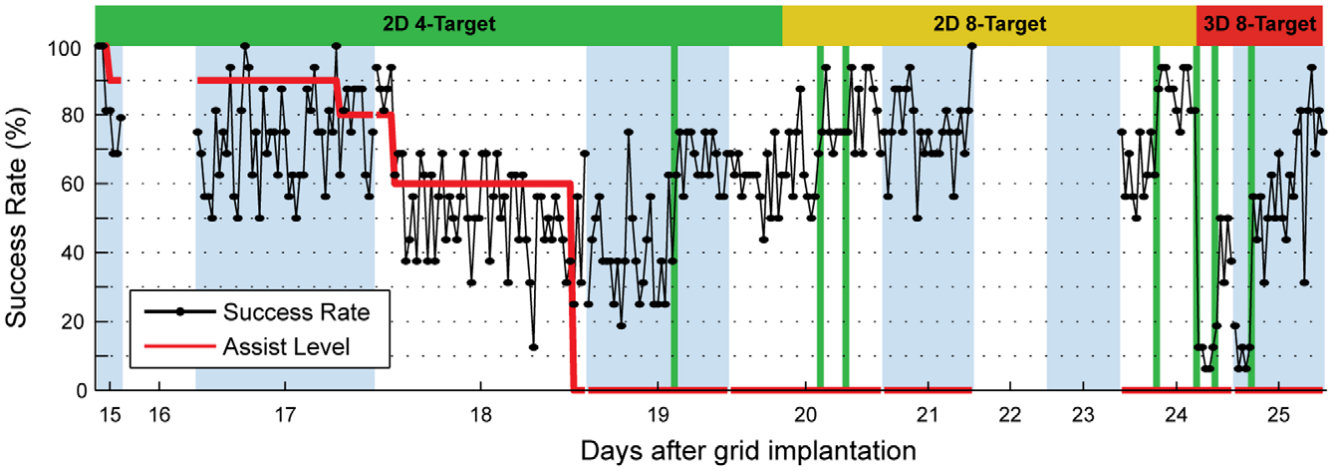
BCI control performance across days. BCI control success rate and computer assist level over time. Success rates are shown for 16-trial blocks of brain control. Alternating white and light-purple zones mark individual days, while vertical green lines mark the occurrence of neural decoder adaptation. Days 16, 22, and 23 were planned days off.

**Figure 3:**
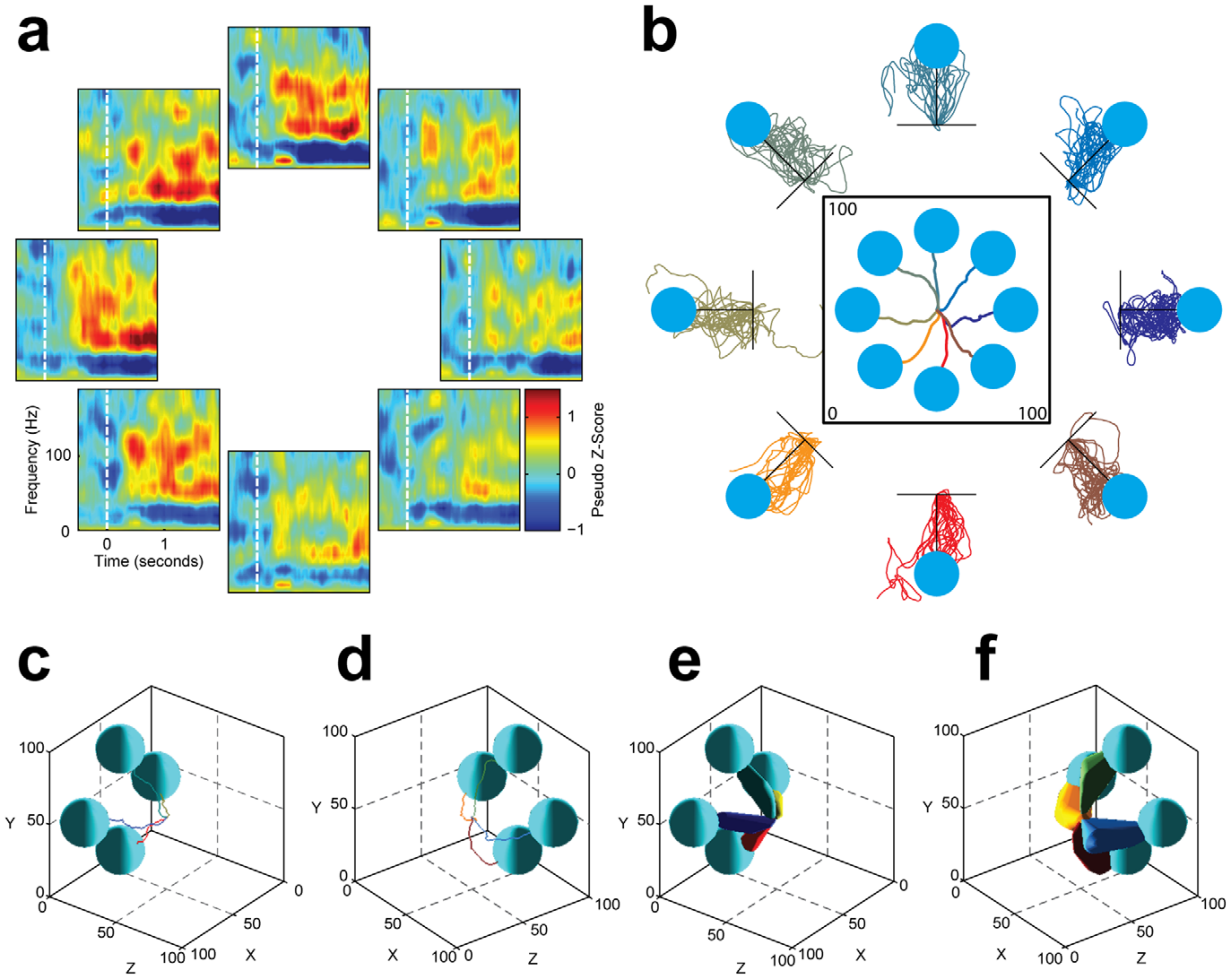
ECoG signal modulation and brain-controlled cursor movement trajectories during 2D (176 trials) and 3D (80 trials) cursor movements. (a) Time-frequency plots of Channel 4 for eight targets during 2D cursor movement. Time 0 represents target onset, and the color represents change from baseline. (b) Cursor trajectories averaged over successful trials (center plot) and individual trajectories of all trials during 2D cursor movement. (c, d) Cursor trajectories averaged over successful trials for the front and back targets, respectively, for 3D cursor movement. (e, f) The 95% confidence intervals of cursor trajectories of all trials for the front and back targets, respectively, for 3D cursor movement. For all trajectory plots in this figure, the circles/spheres show the effective target size, i.e. their radii equal the sum of radii of the target and cursor balls. The unit of the x, y, and z-axes is in percentage of the workspace.

**Figure 2** shows the success rate and computer assist level over 11 days of consistent BCI training (Days 15 to 25). Using the turn-taking adaptation scheme, the participant learned to control 2D cursor movement within a week, achieving a success rate of 87% over 176 trials in the last 2D cursor control session (**Movies S1 and S2**; Chance success rate: 8%). **Figure S3** shows the evolution of neural decoding weights over multiple decoder adaptation sessions. While decoding weights were adapted for optimal performance on Days 19, 20, and 24, decoding weights used for real-time BCI control were relatively constant between sessions. **Figure 3A** shows the time-frequency plots of one sample ECoG channel (Channel 4) with strong high-gamma band power increase for the left, upper-left, and top targets. This pattern was as expected since the high-gamma band of this channel increased in power for attempted thumb movement (**Fig. 1C**) and we instructed the participant to attempt thumb movement to drive the cursor leftward and upward. ECoG signal modulation by desired cursor movement direction enabled the OLE decoder to extract cursor velocity control signals from ECoG feature activities. **Figure 3B** shows trajectories of brain-controlled 2D cursor movement. The distance ratio was 1.53±0.66 (mean ± standard deviation), and the movement error was 0.17±0.14. The cursor's percent time at the boundary was 0%. The time to target was 2.05±0.92 sec for successful trials.

### Transition from 2D to 3D Control

Three-dimensional brain control was built upon 2D control using the following two techniques: 1) For the participant, the existing association between attempted movement and 2D cursor movement direction previously learned was preserved while a third attempted movement, wrist flexion/extension, was added to control cursor movement along the z-axis; 2) For the neural decoder, existing decoding weights for the first two dimensions were also preserved, allowing the decoding weights for the third dimension to be gradually blended into the existing set of 2D weights (**Fig. S3**). The participant started with a success rate of approximately 10%, and reached a success rate of 48% after two rounds of neural decoder adaptation spanning two days. Then, with fixed neural decoding weights, the participant rapidly improved his performance, achieving a final success rate of 80% for 3D cursor control (**Fig. 3C–F**; **Movies S3 and S4**; Chance success rate: 0.4%). The distance ratio and movement error were 2.85±1.25 and 0.40±0.28, respectively, and the cursor's percent time at the boundary was 2% for the last block of 80 trials. The time to target for the successful trials was 2.94±1.16 sec. On Day 27, the participant controlled 3D movement of a prosthetic arm successfully hitting physical targets (**Movies S5 and S6**) without computer assist, and he commented that this was the first time that he reached out to another individual in seven years.

## Discussion

ECoG has a spatial scale in-between that of electroencephalography (EEG) and intracortical microelectrode recording, and it has been suggested that ECoG might offer a good balance between spatiotemporal resolution, invasiveness and signal stability for brain-computer interface applications [3], [4], [7], [13]. The current study investigated the feasibility of an ECoG-based BCI in an individual with tetraplegia caused by a complete cervical spinal cord injury seven years prior to grid implantation, and there are two main findings. First, the participant activated neuronal ensembles in the motor and somatosensory cortices with a coordinated spatiotemporal pattern during attempted movement. Spatially, the somatotopic organization was generally preserved, in agreement with previous fMRI studies in individuals with chronic spinal cord injury [34], [35]. Temporally, high-gamma band activity, which presumably represents local neuronal population activity [36], [37], is tightly coupled to attempted arm and hand movement, similar to previous reports of motor cortical neuronal activity recorded with intracortical microelectrode arrays in individuals with tetraplegia [5], [38], [39]. Second, the participant was able to volitionally modulate sensorimotor cortical activity to achieve high-fidelity real-time BCI control of 2D and 3D cursor movement. Previous studies have demonstrated the feasibility of ECoG-based BCI in able-bodied individuals undergoing presurgical brain mapping [7], [12], [40]. The key feature of the current study is that an individual with chronic paralysis was able to achieve reliable BCI control in a very short period after ECoG grid implantation.

We believe that several factors critically contributed to the achievement of 2D and 3D cursor control in the current study. First, we used a high-density ECoG grid (**Fig. 1A**), which offered better spatial resolution than traditional ECoG grids [17], [41]. Second, the current study utilized an online decoder which, given a large set of ECoG signal features, determined the optimal weighting of each feature [16], [42], [43]. This is different from earlier ECoG-based BCI studies where real-time BCI control used only a small number of signal features [4], [7]. Third, our participant progressed very rapidly from 2D to 3D control (**Fig. 2**) because of the unique BCI training scheme, which gradually blended in control for the third dimension while maintaining control for the first two dimensions (**Fig. S3**). This is a potentially useful scheme for incrementally building up control of devices with high degrees of freedom. Fourth, we used the turn-taking adaptation scheme, which alternated the adapting agent between the human subject and the neural decoder for each testing block (∼80 trials) (**Fig. S2**). While one agent was adapting, the other was kept fixed, providing the adapting agent enough time and data to learn its counterpart's behavior. This scheme helped the human subject and the neural decoder quickly converge to an effective set of decoding weights. Last, every day, the BCI experiment started with the previous day's final decoding weights. This scheme is different from previous intracortical microelectrode studies where decoding weights were re-calibrated daily due to changes in the neuronal population. Our scheme likely facilitated incremental learning by the participant from day to day [44].

The current study observed significant high-gamma band activation at the post-central gyrus, with ECoG signals recorded from this area contributing substantially to BCI control as evident in the decoding weights shown in **Figure S3**. Activation of both pre and post-central gyri is often observed in individuals with chronic spinal cord injury during attempted movement [34], [35], [45] and in able-bodied individuals during motor imagery in the absence of overt movement [40], [46]–[48]. Such somatosensory cortical activity may represent efferent copies of motor control signals [46], [49], [50], or reflect engagement of sensory imagery [45].

The current study was limited by its short duration, the fact that a single participant was tested, and the relatively arbitrary association between attempted movement and desired cursor movement direction. It is worth investigating BCI control schemes based on natural neural representation of intended movement in ECoG signals [16], [42], [43]. Furthermore, it is possible that better grid placement maximizing coverage of the motor cortex could have improved performance. Nevertheless, we have demonstrated that the somatosensory cortex can be used to generate BCI control signals, an intriguing finding worthy of further study [51]. Finally, the current study did not measure head and neck electromyography (EMG). However, we are confident that EMG did not contribute to BCI control because the control signals were derived from high-gamma band activities that were typically over the 40–180 Hz range, temporally associated with a decrease in sensorimotor rhythm, and spatially consistent with the somatotopic organization of motor cortex (**Fig. 1**). This agrees with movement-related neurophysiological responses reported by previous studies [52]–[54].

This study demonstrated that an individual with tetraplegia could reliably operate an ECoG-based BCI system to control 3D cursor movement. The promise of this technology lies in the likelihood that the recorded signals will remain robust over the long-term [16], [55], [56] with relatively low hardware and software requirements. Further development of decoding algorithms, BCI user training approaches, and fully-implantable devices with telemetry [57] will allow for longer studies with more participants, which will facilitate the translation of this technology to clinical use.

## Supporting Information

Text S1 **Supplementary notes and references.**
(PDF)

Figure S1 **Overall progression of the BCI experiments.**
(TIFF)

Figure S2 **BCI control and neural decoder training schemes.** (**a**) The participant was instructed to associate desired cursor movement direction with attempted hand, wrist and/or elbow movement to generate cortical activity modulated by desired cursor movement direction. An OLE decoder was trained to directly predict desired cursor velocity signals from cortical activity. (**b**) Flow of a typical BCI experiment session and the turn-taking adaptation scheme. There were 16 trials per block. Each experiment session always started with the last set of decoding weights used in the previous session.
(TIFF)

Figure S3 **Evolution of neural decoding weights over seven decoder adaptation sessions as represented by the vertical green lines in** **Figure 2** **.** This includes the addition of decoding weights for the third dimension starting from the 5^th^ adaptation session. The decoding weight plots are arranged according to the electrode layout on the ECoG grid (Fig. 1A). For each plot, the top, middle, and bottom panels show weights of 40–200 Hz bands for the x (right), y (up), and z (toward the subject) dimensions. Within each panel/dimension, weights for the 40-Hz band are at the top, and weights for the 200-Hz band are at the bottom. The dashed lines separate the plots into seven neural decoder adaptation sessions, with each session containing five blocks of neural decoder adaptation. The final decoding weights were generally consistent with what would be expected based on cortical activity patterns during the motor screening task and the association between attempted movements and desired cursor movement directions. For example, ECoG signal features from electrodes located above the hand area, such as Channels 4 and 5, had negative weights for cursor movement along the x-axis, meaning that when these features were active they would drive the cursor to the left.
(TIFF)

Movie S1 **Brain control of 2D cursor movement.** This movie was recorded when the participant controlled 2D cursor movement using ECoG signals in our research lab. It shows a block of 16 consecutive trials, and the participant hit all 16 targets successfully. Re-published with permission from UPMC (University of Pittsburgh Medical Center).
(MP4)

Movie S2 **Brain control of 2D cursor movement (reconstructed).** This movie is a replay of brain-controlled 2D cursor movement reconstructed from the saved cursor position data for the 16 trials shown in Movie S1.
(MP4)

Movie S3 **Brain control of 3D cursor movement.** This movie was recorded when the participant controlled 3D cursor movement using ECoG signals in our research lab. It shows a block of 16 consecutive trials, and the participant hit 15 out of 16 targets successfully. The 3D virtual environment was rendered on a 3D LCD TV, and the participant wore a pair of 3D glasses to view the 3D scene. Re-published with permission from UPMC (University of Pittsburgh Medical Center).
(MP4)

Movie S4 **Brain control of 3D cursor movement (reconstructed).** This movie is a replay of brain-controlled 3D cursor movement reconstructed from the saved cursor position data for the 16 trials shown in Movie S3.
(MP4)

Movie S5 **Brain control of 3D prosthetic arm movement (hitting targets).** This movie was recorded when the participant controlled the 3D movement of a prosthetic arm to hit physical targets in our research lab.
(MP4)

Movie S6 **Brain control of 3D prosthetic arm movement (touching hands).** This movie was recorded when the participant controlled the 3D movement of a prosthetic arm to touch hands with another individual in our research lab. Re-published with permission from UPMC (University of Pittsburgh Medical Center).
(MP4)
