# Supplementary material for: An Electrocorticographic Brain Interface in an Individual with Tetraplegia
Source: PLoS One. 2013 Feb 6;8(2):e55344. doi: 10.1371/journal.pone.0055344 (PMC3566209; doi:10.1371/journal.pone.0055344)
Supplement: Text S1 — Supplementary notes and references. (PDF) [file pone.0055344.s001.pdf]

Supplementary Note 1. Informed consent
Written informed consent was obtained before initiating any research procedures. The participant held a pen in his mouth and provided the written consent.

Supplementary Note 2. Presurgical and surgical procedures
In order to localize the left sensorimotor cortex and guide the grid placement, six weeks prior to the implantation surgery, functional magnetic resonance imaging (fMRI) was conducted while the participant observed and attempted right hand and arm movements. fMRI data showed that hand and arm areas spanning the precentral gyrus, central sulcus, and postcentral gyrus of the left hemisphere were activated by attempted movement. Both structural and functional MRI data were loaded into the surgical navigation system (Brainlab AG, Feldkirchen, Germany) to guide the grid implantation surgery, and the high-density ECoG grid was placed to cover these areas while avoiding large cortical vessels. The two leads were tunneled down to the chest and exited the skin below the left clavicle. The exit site was covered by sterile dressing, which was changed weekly.

Supplementary Note 3. ECoG electrode localization
The 3D cortical surface of the subject's brain was reconstructed using pre-operative structural MRI images with the Freesurfer image analysis software [1,2]. Initial estimates of ECoG electrode locations were made using post-operative head x-ray images (both anterior-posterior and lateral views) [3]. These locations were further adjusted based on post-operative computed tomography (CT) images, coordinates of exposed electrodes recorded by the surgical navigation system (Brainlab AG, Feldkirchen, Germany) during the grid implantation surgery, grid geometry, and consultation with the neurosurgeon. The electrode locations were then plotted on the subject's 3D cortical surface reconstructed from Freesurfer (**Fig. 1b**). These estimated electrode locations were independently confirmed using the electrode localization method described in [4] based on the pre-operative structural MRI and post-operative CT images.

Supplementary Note 4. Overall study timeline
The testing dates and locations were determined for both the convenience of the participant and the need of the research study. This manuscript uses days after implantation to report the time of relevant events, and Day 0 was the implantation day and Day 28 was the explantation day. The first testing took place at the participant's home on Day 2. Days 2 to 14, the research team conducted extensive screening tasks with the study participant to identify ECoG signal modulation by various attempted hand and arm movements, as well as preliminary BCI training without explicit instruction of associating cursor movement direction with specific attempted movement. Testing on Days 15 to 25 generated consistent BCI control data, which formed the basis of this manuscript. On Day 26, we briefly experimented with other control strategies. On Day 27, preliminary prosthetic arm control experiments were performed, and the ECoG grid was explanted on Day 28.

Supplementary Note 5. Decoding weights calculation
For convenience of discussion, Equations 1 and 2 in the Materials and Methods section are repeated here with subscripts denoting the sizes of vectors and matrices. Here we discuss one typical scenario where 16 trials of BCI control data were used as the training data to calculate the

decoding weights for 3D cursor movement control. A trial was one continuous cursor movement from the center of the workspace to a given target.

$$\hat{v}_{[1\times3]} = f_{[1\times448]}W_{[448\times3]} \quad (1)$$

$$W_{[448\times3]} = (F_{[16\times448]})^{+}V_{[16\times3]} \quad (2)$$

$\hat{v}$: A vector representing the real-time 3D cursor velocity control signal.

$\boldsymbol{f}$: A vector containing the instantaneous activities of all 448 ECoG signal features.

W: A matrix containing 3D decoding weights for all 448 features.

F: A matrix containing activities of 448 features for the 16 trials of training data. Each row contains 448 features' activities averaged over time for each trial.

V: A matrix containing desired cursor movement direction data in 3D space for all 16 trials of training data. Each row contains desired cursor movement data averaged over time for each trial.

$^{+}$: Pseudo-inverse of a matrix.

There are several discussion points:

1) Although decoding weights were calculated using time-averaged data, these weights were applied to instantaneous feature activities ($\boldsymbol{f}$) in Equation 1 to generate the instantaneous cursor velocity control signal ($\hat{v}$) every 33ms [5]. Furthermore, the resultant cursor velocity control signal was proportional and continuous in 3D space, i.e. the participant was able to use ECoG signals to control both the direction and speed of cursor movement in real-time.
2) To calculate the decoding weights (W) using 16 trials of training data with the OLE algorithm [6], we seek a solution to the following equation

$$V_{[16\times3]} = F_{[16\times448]}W_{[448\times3]} \quad (3)$$

   where V, F, and W are the same matrices as in Equation 2. Note that in this case the pseudoinverse of F is underdetermined, and thus the minimum norm solution for F^{+} is used to solve for the final decoding weights (W) in Equation 3.
3) The decoding method used in the current study (Equation 1) enabled continuous and proportional control of the cursor in 2D or 3D space. Here, we use the term "continuous" to indicate that the cursor velocity command can be an arbitrary vector in 2D or 3D space. "Proportional" indicates that the cursor speed will change in proportion to the overall strength of neural feature activity, e.g. the cursor speed will double if all feature activities double at the same time (Equation 1). This is different from the ECoG signal classification approach used by several earlier ECoG studies, where the BCI control signal can only take on one value from a discrete set of options [7-9], e.g. move vs. rest, or move the thumb vs. move the index finger.
4) The initial neural decoding weights were calculated using the first 40 trials of Day 15. In those trials, the cursor was moved automatically by the computer toward the target and the participant attempted thumb and elbow movements in association with cursor movement direction.
5) Decoding weights trained for 3D cursor control were directly applied to 3D endpoint (approximately center of the palm) control of the prosthetic arm on Day 27.

Supplementary Note 6. BCI control strategy

The current study used a control strategy where the participant was instructed to associate desired cursor movement with attempted movement of the thumb, wrist, and elbow. With this scheme and our neural decoder described in **Supplementary Note 5**, the participant was able

move to the computer cursor in 2D and 3D space in arbitrary directions. Here we use a simplified example that only involves two ECoG signal features, f_{thumb} and f_{elbow}, to illustrate the basic idea of the control scheme. f_{thumb} varies between 0 (no attempted thumb movement) and 1 (strong attempted thumb movement), and f_{elbow} also varies between 0 (no attempted elbow movement) and 1 (strong attempted elbow movement).
The control scheme can be expressed as:

$$x = f_{elbow} - f_{thumb} \qquad \text{Eq. A1}$$

$$y = f_{elbow} + f_{thumb} - 1 \qquad \text{Eq. A2}$$

$$(0 \le f_{thumb} \le 1, \quad 0 \le f_{elbow} \le 1)$$

where x and y are horizontal and vertical cursor velocities. Intuitively, the difference between f_{elbow} and f_{thumb} controls cursor horizontal velocity, and the sum of f_{elbow} and f_{thumb} controls cursor vertical velocity. Note an offset value of '-1' was included in Eq. A2 to allow the cursor move both upward and downward. The table below listed f_{thumb} and f_{elbow} activities and the resulting cursor movement:

f_{thumb}	f_{elbow}	$x = f_{elbow} - f_{thumb}$	$y = f_{elbow} + f_{thumb} - 1$	*Cursor movement*
1	*1*	*0*	*1*	*Up*
0	*0*	*0*	*-1*	*Down*
1	*0*	*-1*	*0*	*Left*
0	*1*	*1*	*0*	*Right*
0	*0.5*	*0.5*	*-0.5*	*Right-and-down*
0.5	*0.5*	*0*	*0*	*No movement*

As shown in the above table, to move the cursor in the right-and-down direction, the participant needs to moderately attempt elbow movement. Additionally, our control strategy requires that the participant use attempted movements to generate appropriate high-gamma band activity to keep the cursor speed at zero. If the participant is at rest, the decoder will cause the cursor to move downward in the 2D task or downward and away in the 3D task. The participant did not practice the control strategy outside the experiment sessions.

Supplementary Note 7. The turn-taking adaptation scheme for BCI training
The current study introduced the turn-taking adaptation paradigm for BCI training (**Fig. S2**). Here are a few key points regarding this technique:

1) It was a cooperative process involving two agents, the computer (i.e. the neural decoder) and the human subject. Both agents participated in this process and worked synergistically to improve the overall BCI performance measured as success rates during 2D and 3D cursor tasks.

2) The turn-taking adaptation scheme alternated the adapting agent, and only one agent was allowed to adapt at a time. This method potentially accelerated the convergence between the two agents.
3) This turn-taking method also enabled the contribution from each agent to be determined as the overall success rate increased. As shown in **Figure 2**, during the 2D cursor task, neural decoder adaptation improved the success rate more than the adaptation/learning by the human subject. On the contrary, during the 3D cursor task, it was evident that the participant's adaptation contributed more than neural decoder adaptation since the success rate kept increasing with fixed decoding weights. This is an interesting phenomenon that requires further study.

References

1. Dale AM, Fischl B, Sereno MI (1999) Cortical surface-based analysis. I. Segmentation and surface reconstruction. Neuroimage 9: 179-194.
2. Fischl B, Sereno MI, Dale AM (1999) Cortical surface-based analysis. II: Inflation, flattening, and a surface-based coordinate system. Neuroimage 9: 195-207.
3. Miller KJ, Makeig S, Hebb AO, Rao RP, denNijs M, et al. (2007) Cortical electrode localization from X-rays and simple mapping for electrocorticographic research: The "Location on Cortex" (LOC) package for MATLAB. J Neurosci Methods 162: 303-308.
4. Hermes D, Miller KJ, Noordmans HJ, Vansteensel MJ, Ramsey NF (2010) Automated electrocorticographic electrode localization on individually rendered brain surfaces. J Neurosci Methods 185: 293-298.
5. Wang W, Chan SS, Heldman DA, Moran DW (2007) Motor cortical representation of position and velocity during reaching. J Neurophysiol 97: 4258-4270.
6. Salinas E, Abbott LF (1994) Vector reconstruction from firing rates. J Comput Neurosci 1: 89-107.
7. Kubanek J, Miller KJ, Ojemann JG, Wolpaw JR, Schalk G (2009) Decoding flexion of individual fingers using electrocorticographic signals in humans. J Neural Eng 6: 66001.
8. Wang W, Degenhart AD, Collinger JL, Vinjamuri R, Sudre GP, et al. (2009) Human motor cortical activity recorded with micro-ECoG electrodes during individual finger movements. IEEE EMBC. Minneapolis, MN, 2009.
9. Yanagisawa T, Hirata M, Saitoh Y, Kishima H, Matsushita K, et al. (2011) Electrocorticographic control of a prosthetic arm in paralyzed patients. Ann Neurol.
