# Supplementary figures and images for: An Electrocorticographic Brain Interface in an Individual with Tetraplegia
Source: PLoS One. 2013 Feb 6;8(2):e55344. doi: 10.1371/journal.pone.0055344 (PMC3566209; doi:10.1371/journal.pone.0055344)

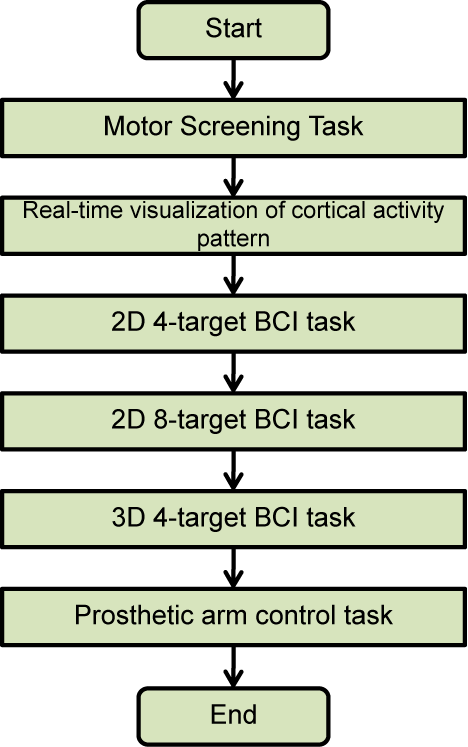

Supplement: Figure S1 — Overall progression of the BCI experiments. (TIFF) [file pone.0055344.s002.tiff]

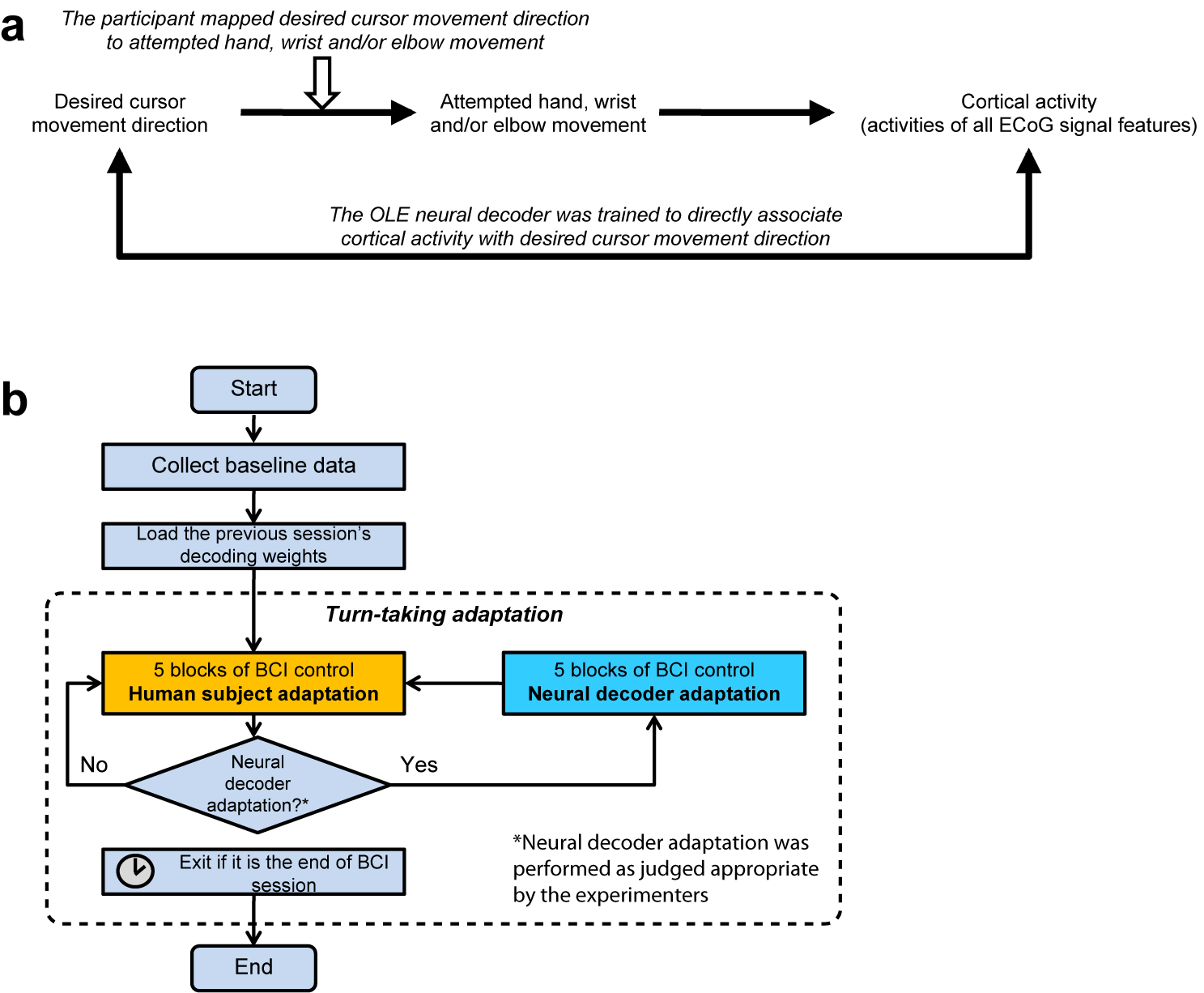

Supplement: Figure S2 — BCI control and neural decoder training schemes. (a) The participant was instructed to associate desired cursor movement direction with attempted hand, wrist and/or elbow movement to generate cortical activity modulated by desired cursor movement direction. An OLE decoder was trained to directly predict desired cursor velocity signals from cortical activity. (b) Flow of a typical BCI experiment session and the turn-taking adaptation scheme. There were 16 trials per block. Each experiment session always started with the last set of decoding weights used in the previous session. (TIFF) [file pone.0055344.s003.tiff]

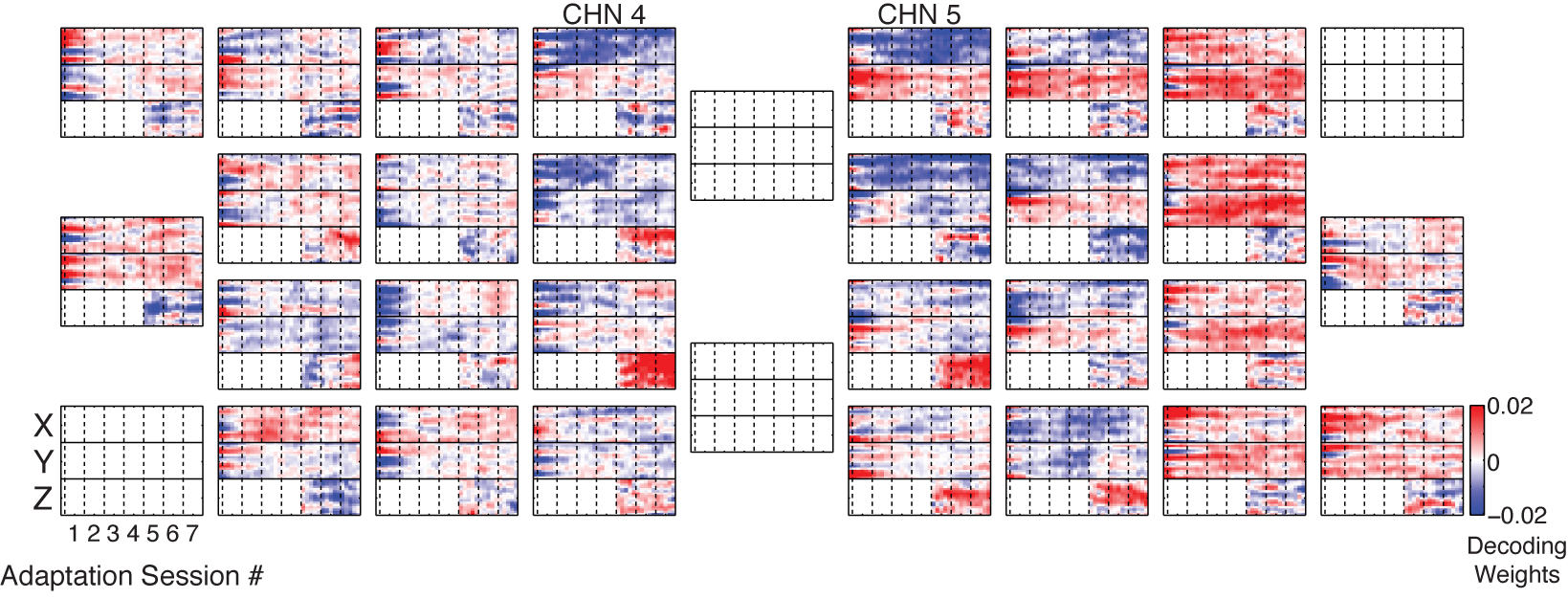

Supplement: Figure S3 — Evolution of neural decoding weights over seven decoder adaptation sessions as represented by the vertical green lines in Figure 2 . This includes the addition of decoding weights for the third dimension starting from the 5th adaptation session. The decoding weight plots are arranged according to the electrode layout on the ECoG grid (Fig. 1A). For each plot, the top, middle, and bottom panels show weights of 40–200 Hz bands for the x (right), y (up), and z (toward the subject) dimensions. Within each panel/dimension, weights for the 40-Hz band are at the top, and weights for the 200-Hz band are at the bottom. The dashed lines separate the plots into seven neural decoder adaptation sessions, with each session containing five blocks of neural decoder adaptation. The final decoding weights were generally consistent with what would be expected based on cortical activity patterns during the motor screening task and the association between attempted movements and desired cursor movement directions. For example, ECoG signal features from electrodes located above the hand area, such as Channels 4 and 5, had negative weights for cursor movement along the x-axis, meaning that when these features were active they would drive the cursor to the left. (TIFF) [file pone.0055344.s004.tiff]
